# To assess implementation of trust policy (smoke free policy) on an acute mixed mental health ward setting

**DOI:** 10.1192/bjo.2021.247

**Published:** 2021-06-18

**Authors:** Antigoni Elisseou, Saika Rahuja

**Affiliations:** Greater Manchester Mental Health NHS Foundation Trust

## Abstract

**Aims:**

To assess implementation of Trust Policy (Smoke Free Policy) on the acute adult mental health unit To evaluate barriers to implementation of local standards and NICE guidelines To evaluate if Q-Risk score is being calculated and noted.

**Background:**

There are about 34,000 people residents in mental health facilities in England and Wales on any one day (Commission for Healthcare Audit and Inspection 2005) and many of them smoke.

Smoke free policy implemented in the GMMH since 1st of July 2018.

Smoking is single largest preventable cause of ill health & premature mortality in England.

Smoking prevalence is significantly higher among people admitted to hospital due to the mental illness i.e. 70%

According to WHO SHS (second hand smoking), is a human carcinogen to which there is no safe level.

**Method:**

An audit tool questionnaire was used to collect the data on the Acute Mixed mental health ward setting i.e. Bronte Ward, Laureate House, Wythenshawe Hospital

Identified method: interview with each patient, PARIS documentation review and Patient's Kardex review.

Sample size: 23 and on re-audit 12.

Method of data input: Microsoft Excel

Data were analyzed by calculating percentage

**Result:**

The majority of the patients that took part in the Audit were smokers (91%), a high percentage overall. This indicate how important it is for a plan to be in place regarding smoking on the ward since there is a smoke free policy now in the GMMH. Our results showed that not everyone was asked regarding their smoking status (87%).

An important figure that came out from the results was that only 50% of the patients asked about their smoking status were told that there is a smoke free policy.

For a smoke free policy ward only 33% of the smokers that took part in the audit were provided with brief advice regarding smoking cessation which shows that there might be a need of a more precise implementation regarding support to receive brief intervention for smoking cessation, NRT and specialist advice.

The results also showed that the QRisk is not calculated, a useful marker of cardiovascular risk.

**Conclusion:**

Give leaflets regarding smoking cessation on admission, offer support and advice to all the patients being on the ward. And re-audit in due course to see the effect of this intervention.

